# Association of Hospital-Based Social Needs Interventions with Potentially Preventable Admissions

**DOI:** 10.1007/s11606-024-09203-w

**Published:** 2025-01-17

**Authors:** Margae J. Knox, Amanda L. Brewster, Jennifer Ahern, Stephen M. Shortell, Emily L. Hague, Hector P. Rodriguez

**Affiliations:** 1 Kaiser Permanente Division of Research, Pleasanton, CA USA; 2 School of Public Health, Division of Health Policy and Management, University of California, Berkeley, Berkeley, CA USA; 3 School of Public Health, Division of Epidemiology, University of California, Berkeley, Berkeley, CA USA; 4 Mathematica Policy Research, Oakland, CA USA

**Keywords:** health-related social needs, hospitals, meal delivery, transportation, violence prevention, mobile clinics, preventable admissions, avoidable admissions, quality, population health

## Abstract

**Background:**

External incentives increasingly encourage hospitals to address health-related social needs, yet limited evidence exists about whether social needs interventions are associated with quality indicators like potentially preventable admissions.

**Objective:**

We analyze whether four hospital interventions—meal delivery, transportation to health services, mobile clinics, and community-oriented violence prevention programs—are associated with potentially preventable hospitalizations.

**Design:**

Cross-sectional analysis of survey-based and claims-based data.

**Participants:**

In total, 813 hospitals from 14 states, representing 6,003,739 adult all-payer hospital admissions.

**Approach:**

This study merged 2017 Healthcare Cost Utilization Project State Inpatient Databases with 2017 American Hospital Association survey data. Generalized linear models for each of the four interventions were separately estimated to assess the association with potentially preventable hospitalizations, controlling for hospital and patient characteristics. Sensitivity analyses restricted regression modeling to adult Medicaid and Medicare beneficiaries.

**Key Results:**

A minority (13%) of hospital admissions were potentially preventable. 24% of hospitals offered transportation to health services, 16% offered mobile clinic services, 16% offered violence prevention programs, and 9% offered meal delivery. In adjusted analyses, hospital meal delivery was associated with 1.1% lower predicted probability of a potentially preventable hospitalization (95% confidence interval (CI) −2.1% to −0.1%), with a stronger relationship among Medicaid beneficiaries (−2.3%, 95% CI −3.5% to −1.0%). Associations for other social needs interventions were not statistically significant.

**Conclusions:**

Hospital meal delivery was associated with significantly lower probability of potentially preventable hospitalizations, with larger effects for Medicaid beneficiaries. Meal delivery may support hospital quality. More nuanced understanding about the reach of social needs interventions is needed to further examine the impact of these hospital-based services on patient outcomes.

**Visual Abstract:**

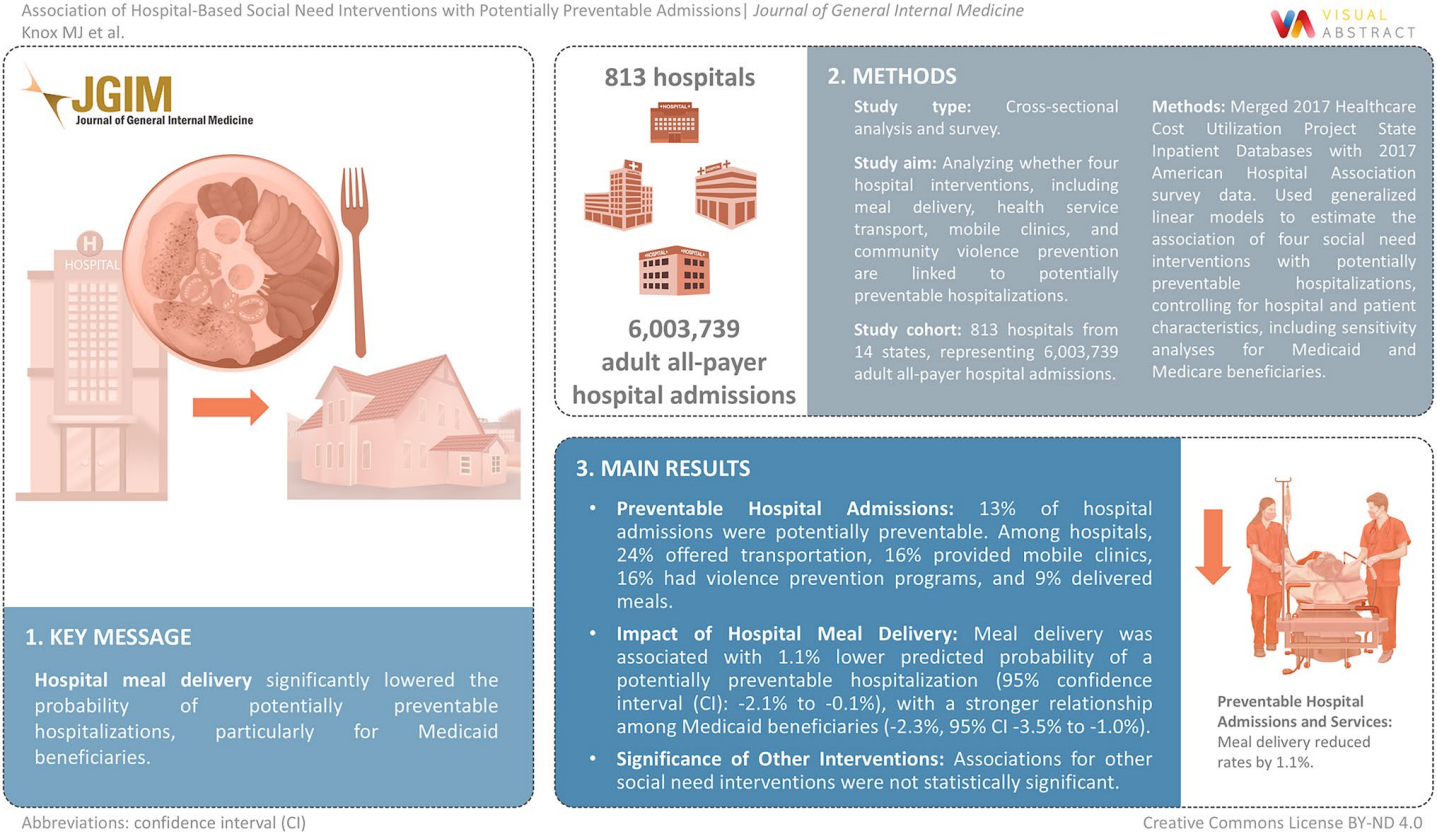

**Supplementary Information:**

The online version contains supplementary material available at 10.1007/s11606-024-09203-w.

## INTRODUCTION

As value-based healthcare reforms evolve, scrutiny of hospital quality and accountability for population health is increasing.^1,2^ Simultaneously, a growing evidence base links health-related social needs to worse health outcomes and greater hospital and emergency department use.^3–6^ Thus, new Centers for Medicare and Medicaid Services (CMS) guidance encourages health systems to implement cost-effective, innovative strategies to address health-related social needs,^7^ building on 2019 National Academy of Medicine recomendations.^8^ Further, quality measures that began in 2023 are assessing the extent to which hospitalized patients are screened and offered assistance for health-related social needs.^9–11^

In addition to the recent CMS guidance and new quality measures, pre-existing policies uniquely position hospitals to offer social needs interventions, including the CMS hospital readmissions reduction program^12^ and Delivery System Reform Incentive Payment (DSRIP) programs for hospitals serving largely Medicaid and uninsured patients.^13,14^ Further, non-profit hospitals are required by law to demonstrate investment in community benefits.^15–17^ While past research has identified hospital characteristics associated with social needs activities,^18,19^ highlighting strong connections between hospital social needs activities and participation in value-based payment models like accountable care organizations, similar national data has not been used to establish relationships between specific hospital-based social needs interventions and quality or population health outcomes.

To advance evidence, we examined the relationship of hospital use of social needs interventions for meal delivery, transportation to health services, mobile clinics, and community-oriented violence prevention programs with potentially preventable admissions. Prior studies on social needs interventions have promising results. Outcomes associated with meal delivery, the most extensively researched intervention, include fewer emergency department (ED) visits and hospitalizations,^20^ shorter length of stay,^21^ lower total expenditures,^22,23^ and lower post-hospitalization mortality.^24,25^ Nonemergency medical transportation interventions have been found to improve appointment attendance rates, though evidence on subsequent utilization is limited.^26^ Mobile clinics have been associated with avoided ED visits and decreased hospital length of stay.^27^ Violence prevention programs in the community have been found to increase quality-adjusted life years and save costs in one study, though other evaluations have found no impact.^28^ However, prior literature across these interventions has not assessed hospital use of these services with potentially preventable hospitalizations, a widely used quality measure that reflects timely and effective care coordination across the hospital, outpatient, and community settings.^29^

**Figure. 1:**
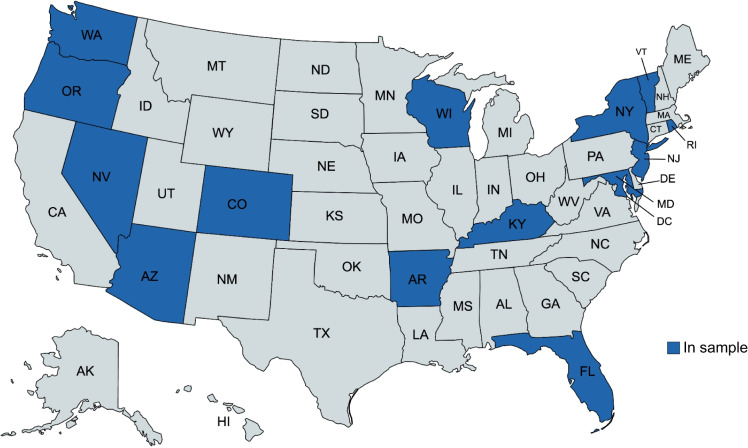
States represented in the analytic sample.

Our study makes a unique contribution by integrating American Hospital Association (AHA) annual survey responses with the Health Care Cost and Utilization Project State Inpatient Databases (HCUP-SID) to empirically assess the extent to which hospital-based social needs interventions among US hospitals are related to potentially preventable hospitalizations, an underexamined outcome. Our study analyzes a large sample of 6,003,739 adult hospital admissions from 813 hospitals in 14 states (Fig. 1). We hypothesized that each of the social needs interventions would be associated with lower odds of having a potentially preventable hospitalization.

## METHODS

### Study Design

We conducted a cross-sectional analysis of 2017 American Hospital Association (AHA) annual survey responses linked to 2017 Healthcare Cost and Utilization Project State Inpatient Databases (HCUP-SID) admissions data for 14 US states: Arkansas, Arizona, Colorado, Florida, New Jersey, New York, Oregon, Rhode Island, Vermont, Washington, Kentucky, Maryland, Nevada, and Wisconsin. States were selected based on the state’s HCUP-SID availability, quality of data available, and ability to link AHA and HCUP-SID data. States were also chosen to represent diverse geographic, urban/rural, and policy environments. Admissions include all payers including Medicare, Medicaid, private insurance, and others.

The AHA annual survey is a voluntary survey of all hospitals in the USA and has a response rate at or near 80%.^34^ We restricted analyses to all general medical and surgical hospitals with complete responses to AHA survey questions about social needs–related services, resulting in 6,003,739 adult inpatient admissions across 813 hospitals. This study was approved by the University of California, Berkeley, Office for the Protection of Human Subjects.

### Measures

#### Outcome Variable

The primary outcome is whether a patient’s hospitalization was potentially preventable or not. Potentially preventable admissions were defined using AHRQ’s Prevention Quality Indicators composite,^29^ which classifies potentially preventable admission based on ten primary diagnoses where the admission may have been avoided through high-quality care coordination. Diagnoses include diabetes complications, chronic obstructive pulmonary disease (COPD), asthma, hypertension, heart failure, community-acquired pneumonia, and urinary tract infection.

#### Independent Variables

The main independent variables are four hospital-based social needs interventions—meal delivery, transportation to health services, mobile clinics, community-oriented violence prevention programs—assessed based on self-reported AHA survey responses. We consider all respondents reporting hospital ownership or provision of a given health-related social needs intervention in the 2017 AHA survey to be adopters of the intervention (see survey question wording Appendix 6). Each intervention was analyzed separately because the internal consistency reliability for a 4-item composite measure was low (Cronbach’s alpha, $\alpha$ = 0.45), suggesting that hospitals do not implement social needs interventions as a part of a cohesive strategy. Evidence specific to each intervention may also be more actionable than results based on a composite score.

#### Covariates

Hospitalization-level covariates are based on HCUP-SID admissions data and include variables that we hypothesized would be associated with hospital use of social needs interventions and quality of care, including patient sex, age (19–39, 40–64, or 65+), primary payer (private insurance, Medicare, Medicaid, or other), race (White, Black/African American, Hispanic, Asian/Pacific Islander, or other), zip-code income quartile, and Charlson Comorbidity Index (0, 1, 2, or 3+ conditions). For patients dually eligible for Medicare and Medicaid, HCUP-SID methodology categorizes the primary payer as Medicare in most cases.^35^ Hospital covariates are based on AHA survey responses and include ownership (public, nonprofit, for-profit), size (0–99 beds, 100–299 beds, 300+ beds), belonging to a health system, participating in an accountable care organization, participating in bundled payment, and rurality (metropolitan, micropolitan, or rural).

### Statistical Analysis

The association between each social needs intervention and potentially preventable hospitalizations was assessed using separate generalized linear models with a logit link for binomial outcomes and clustering by hospital.

We calculated separate stabilized inverse propensity of treatment weights (IPTW) for each model to address selection bias, as hospitals may be more likely to implement a social needs intervention given their patient population characteristics. Weights were constructed based on all patient characteristics (sex, age, race, payer, zip code–level income, and comorbidity index) and predict the likelihood that a patient will receive care at a hospital where the social needs intervention is used. Prior to weighting, for example, patients in the highest income quartile zip codes more commonly received care at hospitals that had implemented meal delivery, transportation to health services, and community-oriented violence prevention programs. We assessed whether propensity scores for each independent variable shared common support and found full overlap, i.e., no observations outside the region of common support.

Final models incorporated IPTW weights and adjusted for both patient level and hospital level covariates. In postestimation, we assessed the variance inflation factor (VIF) to ensure models were not impacted by multicollinearity and confirmed that each categorical variable level was below the common threshold of 10 for indicating high inflation.^36^ All results are reported as marginal effects, or the percentage point change in probability of a potentially preventable hospital admission.

### Sensitivity Analyses

In sub-analyses, we also examined results specific to Medicare beneficiaries and specific to Medicaid beneficiaries. We anticipated that the presence of social needs interventions may have a stronger influence among Medicare and Medicaid beneficiaries compared to private-pay beneficiaries due to greater health-related social needs among low-income and elderly populations.^37^ We also anticipate that the CMS leadership in value-based contracting may have prompted more robust implementation of social needs interventions for Medicare and Medicaid beneficiaries. In addition, for interventions significantly associated with potentially preventable admissions, we examine related specific PQI composites in post hoc analysis to better understand if the intervention is associated with particular reasons for admission.

## RESULTS

Across the 813 hospitals, the median number of patient admissions was 4836 (interquartile range 982–11,077, range 34–99,292). A minority (13.3%) of hospital admissions were classified as potentially preventable. 8.6% of hospitals (*n* = 69) offered meal delivery, 24.1% (*n* = 196) offered transportation to health services, 16.2% (*n* = 132) offered mobile clinics, and 16.4% (*n* = 133) offered community-oriented violence prevention programs. More than half of hospitals (55%, *n* = 449) had not implemented any of the four social needs interventions, and 30% (*n* = 243) had implemented one intervention only. Just 7 hospitals (1%) had implemented all 4 interventions (see intervention combinations, Appendix Table 4).

Most hospitals (78.4%) were not-for-profit. 70.1% of hospitals were members of a larger health system, 7.8% were academic medical centers, 44.3% were accountable care organization members, and 25.2% participated in bundled payments. 67.6% were in metropolitan areas while 17.0% were rural hospitals (Table 1). Hospital descriptive characteristics based on provision of each social risk intervention are available in Appendix Table 1. Provision was more common among non-for-profit owned hospitals across all interventions, and more common among larger hospitals, teaching hospitals, hospitals in an ACO or participating in bundled payments, and hospitals in metropolitan areas for all interventions except meal delivery.

**Table 1:** Hospital Characteristics from 14 US States

Hospital covariate	*N* (total = 813)	%
Ownership		
Public (county, state, other gov’t)	111	(13.7%)
Not-for-profit (non-gov’t)	637	(78.4%)
For-profit	65	(8.0%)
Bed size (small, medium, large)		
Up to 99 beds	322	(39.6%)
100 to 299 beds	283	(34.8%)
300+ beds	208	(25.6%)
Member of larger health system	570	(70.1%)
Teaching hospital	63	(7.8%)
Participates in an ACO	360	(44.3%)
Participates in bundled payment	205	(25.2%)
Rurality		
Metro	550	(67.6%)
Micro	125	(15.4%)
Rural	138	(17.0%)

Just over half of patients (51.2%) were female. 49.6% were age 65 or older while 37.2% were 40 up to age 65. Over two-thirds of patients (68.3%) were White, 14.5% were Black/African American, 10.0% were Hispanic, 2.2% were Asian or Pacific Islander, and 5.1% were another race/ethnicity. One-third (33.2%) had three or more comorbidities while 28.8% had none. Hospital size included small (up to 99 beds, 39.6%), medium (100–299 beds, 34.8%), and large (300+ beds, 25.6%) capacity. The payer mix included 54.0% Medicare, 16.5% Medicaid, 23.2% private insurance, and 6.3% other payer (e.g., self-pay, no charge). Patient characteristics for the potentially preventable admissions compared to admissions that were not potentially preventable differed across all characteristics at a statistically significant *p*-value level <0.001 (see Table 2). After weighting, observed patient characteristics were balanced across hospitals with and without social needs interventions, particularly for pre-weighting differences in patients’ zip code–based income quartile (see Appendix Table 2).

**Table 2:** Patient Characteristics, by Potentially Preventable Hospitalization Status

	Total		Not potentially preventable admission		Potentially preventable admission		
	*N*=6,003,739	%	*N*=5,207,668	%	*N*=796,071	%	*p-*value
Sex (female)	3,071,941	(51.2%)	2,647,539	(50.8%)	424,402	(53.3%)	***
Age categories							***
19 to <40	789,872	(13.2%)	730,860	(14.0%)	59,012	(7.4%)	
40 to < 65	2,234,405	(37.2%)	1,979,347	(38.0%)	255,058	(32.0%)	
65+	2,979,462	(49.6%)	2,497,461	(48.0%)	482,001	(60.5%)	
Race							***
White	4,102,284	(68.3%)	3,578,229	(68.7%)	524,055	(65.8%)	
Black	867,843	(14.5%)	723,943	(13.9%)	143,900	(18.1%)	
Hispanic	598,180	(10.0%)	518,625	(10.0%)	79,555	(10.0%)	
Asian or Pacific Islander	131,810	(2.2%)	117,086	(2.2%)	14,724	(1.8%)	
Native American	28,771	(0.5%)	24,975	(0.5%)	3796	(0.5%)	
Other	274,851	(4.6%)	244,810	(4.7%)	30,041	(3.8%)	
Charlson Comorbidity Index							***
0 comorbidities	1,729,725	(28.8%)	1,678,360	(32.2%)	51,365	(6.5%)	
1 comorbidity	1,297,421	(21.6%)	1,116,442	(21.4%)	180,979	(22.7%)	
2 comorbidities	983,733	(16.4%)	823,487	(15.8%)	160,246	(20.1%)	
3+ comorbidities	1,992,860	(33.2%)	1,589,379	(30.5%)	403,481	(50.7%)	
Zip code–level median household income (national quartiles)							***
$1–$43,999	1,656,987	(27.6%)	1,403,606	(27.0%)	253,381	(31.8%)	
$44k–$55,999	1,513,193	(25.2%)	1,307,932	(25.1%)	205,261	(25.8%)	
$56k–73,999	1,475,089	(24.6%)	1,292,841	(24.8%)	182,248	(22.9%)	
74k+	1,358,470	(22.6%)	1,203,289	(23.1%)	155,181	(19.5%)	
Primary payer							***
Medicare	3,244,118	(54.0%)	2,718,080	(52.2%)	526,038	(66.1%)	
Medicaid	993,212	(16.5%)	873,759	(16.8%)	119,453	(15.0%)	
Private insurance	1,390,789	(23.2%)	1,280,049	(24.6%)	110,740	(13.9%)	
Other (self, no charge, other, missing)	375,620	(6.3%)	335,780	(6.4%)	39,840	(5.0%)	

^***^Chi-squared test *p*-value < 0.001

**Figure. 2:**
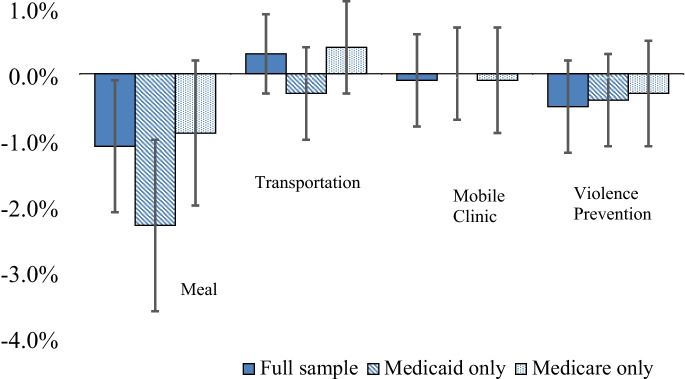
Marginal effects on potentially preventable hospitalization for each social needs intervention.

In adjusted analyses, the marginal effect of hospital meal delivery was a 1.1% lower predicted probability of potentially preventable hospital admission (95% confidence interval (CI) −2.1% to −0.1%). The association was slightly stronger among admissions for Medicaid beneficiaries, with a 2.3% lower (95% CI −3.5% to −1.0%) predicted probability of potentially preventable hospitalization among hospitals providing meal delivery. Among admissions for Medicare beneficiaries, the association between meal delivery and potentially preventable hospitalization was not statistically significant (Table 3/Fig. 2). Associations between meal delivery and chronic condition-only potentially preventable admissions were consistent with the overall analysis, while associations remained statistically significant only for Medicaid beneficiaries when analyzing diabetes-related potentially preventable admissions (Appendix Table 5).

**Table 3:** Marginal Effect for Each Activity to Address Health-Related Social Needs (Percentage Point Change in Probability of Potentially Preventable Admission When Activity Is Present)

GLM models	Overall			Medicaid			Medicare		
	Marginal effect	95% CI LL	95% CI UL	Marginal effect	95% CI LL	95% CI UL	Marginal effect	95% CI LL	95% CI UL
Meal delivery	−1.1	−2.1	−0.1	−2.3	−3.5	−1.0	−0.9	−2.1	0.2
Transportation to medical appointments	0.3	−0.4	0.9	−0.3	−1.0	0.4	0.4	−0.3	1.1
Community-oriented violence prevention programs	−0.5	−0.2	0.2	−0.4	−1.1	0.3	−0.3	−1.1	0.5
Mobile health clinic	−0.1	−0.8	0.6	0.0	−0.7	0.7	−0.1	−0.9	0.7
Extensive partnerships	0.1	−0.5	0.6	0.0	−0.7	0.8	0.0	−0.7	0.7

^*^All models control for gender, age category, race, zip code–level income, comorbidities, payer, ownership, beds, system, teaching, accountable care organization participation, bundled payment participation, and rurality

*CI* confidence interval, *LL* lower limit; *UL* upper limit

There was no association between transportation to medical appointments, community-oriented violence prevention programs, or mobile health clinics with potentially preventable hospitalizations. There was also no association for these interventions in sensitivity analyses restricted to Medicaid and Medicare beneficiaries (Table 3/Fig. 2).

Other characteristics associated with greater risk of potentially preventable admissions included female sex, age over 65 (compared to ages 19–39), one or more comorbidities (compared to none), Black or Hispanic race/ethnicity (compared to White), and micropolitan or rural hospital setting (compared to a metropolitan setting). Characteristics associated with lower risk of potentially preventable admissions included higher patient zip code–level income, larger hospital bed size, and teaching hospital status (see Appendix Table 3).

## DISCUSSION

Our analysis of whether hospital-based social needs interventions are associated with potentially preventable hospitalizations found a small, statistically significant association with providing meal delivery. The association between meal delivery and lower potentially preventable hospitalizations suggests that meal delivery may be a valuable component of efforts to support population health, reducing exacerbation of health conditions. Meal delivery may also support cost savings, as national estimates indicate that potentially preventable hospitalizations account for 33.7 billion in aggregate yearly costs.^38^

Based on prior studies, meal delivery may enhance nutrition through the meals themselves, by improving emotional wellbeing through social connections with those who deliver meals, and by allowing recipients to use limited resources for other needs.^22,39^ In addition, given that meal delivery was least commonly implemented, with less than 9% of hospitals offering meal delivery, it is possible that meal delivery services are an indicator of hospital culture or strategic commitment to addressing social needs.

More generally, multiple frameworks suggest pathways by which hospital-based social needs interventions may improve care quality and population health.^30,31^ Interventions may address competing demands and alleviate stress. They may also strengthen connections to continuous, community-based care, which is important since access to care is traditionally considered a driver of potentially preventable hospitalizations.^32^ In addition, hospital-based social needs interventions could be a mechanism for community building and investment, which is important in light of recent evidence that greater social spending is associated with lower likelihood of potentially preventable hospitalizations.^33^

Nonetheless, there were no associations of hospital-based transportation to health services, mobile clinics, and violence prevention with potentially preventable hospitalizations. One potential explanation is the limited reach of these interventions. For example, it is possible that interventions were offered only to patients with a specific payer or who met narrow eligibility criteria. While some evidence indicates programs are more effective when offered to a narrow population of focus, other work argues that narrow eligibility criteria miss people who could benefit and inhibit broad, population impact.^40^ Alternatively, available interventions may have low uptake, as documented in clinical trials of transportation assistance.^41^ Current screening or needs identification may also be insufficient;^42^ or patients may not find the offered services useful.^43^

Another consideration is that addressing health-related social needs may impact social determinants of health in ways not captured in claims data. For example, community-oriented violence prevention programs may impact social outcomes such as improved return to schooling, boosting employability.^44^ Despite long-term benefits of education, such benefits would not be captured in clinically focused measures such as the potentially preventable hospitalization measure that we examined.

As CMS and others promote investments in social needs interventions, our analysis provides important foundational evidence on relationships between specific social needs interventions and a widely used hospital quality of care measure. There is much evidence for social needs interventions generally, ranging from findings of 11% fewer hospitalizations^46^ and a 69% reduction in hospitalized days^47^ to more mixed outcomes.^45^ Yet many of these studies focus on populations with higher acuity health concerns and assess multi-component, case management-based interventions. Our study adds to existing evidence with its unique population-wide lens and attention to multiple specific social risk interventions.

Given the modest observed relationship between meal delivery and potentially preventable hospitalizations and no observed relationship for other social needs interventions, future studies and subsequent guidance by CMS should more closely examine patient eligibility and intervention reach.^48^ CMS and other quality stakeholders should also promote resources and systems that support innovation capacity to develop and implement social needs interventions including use of patient experience feedback. Prior research has found that receiving value-based payments alone was not associated with implementing social risk screening, though greater organizational innovation capacity was associated.^49^ This suggests that, in spite of external incentives, hospitals may not implement social needs interventions because they lack the internal capacity to appraise and implement them. Innovation capacity may be especially important since new social needs interventions often need to be locally tested and tailored for unique needs of patients rather than directly replicate programs used elsewhere.^50^ Outcomes among organizations that adopt innovations more superficially based on industry trends or regulatory pressures have been found to be less impactful relative to early adopters.^50^

### Limitations

Our findings should be considered in light of certain limitations. Hospital interventions to address health-related social needs are reported dichotomously at the hospital level. Though the AHA survey includes specific definitions for each intervention (see Appendix), hospital implementation and scope of services could entail substantial variability. Intervention reach is also not assessed, and we cannot determine which patients were recently hospitalized or received social needs interventions to understand intervention impacts more directly. As previously noted, future work could more specifically examine these populations to better understand intervention effectiveness.

In addition, potentially preventable hospitalizations are influenced by outpatient care availability and community-based resources, which may dampen the associations of hospital-based efforts to coordinate patient care. A wider range of outcome measures including repeated emergency department visits and readmissions as well as process measures such as community and health network referrals could help elucidate these influences. Last, we cannot draw causal conclusions given the cross-sectional data analyzed. Despite these limitations, our study is the first to examine the association of hospital-based social needs interventions and quality of care based on potentially preventable hospitalization prevalence across multiple states and across all-payers, which advances evidence in important ways.

## CONCLUSION

As hospitals increasingly pursue value-based care, which involve accountability for cost and quality, it is commonly assumed that social needs interventions will help them achieve quality and population health goals. Our results suggest that meal delivery services may be an effective strategy for addressing health-related social needs and ensuring high-quality care for chronic conditions. However, the lack of association for other social needs interventions suggests that greater understanding about the reach and implementation designs of these interventions is needed to accurately assess their potential impact on quality of care. The emerging evidence on social needs interventions is especially valuable as federal agencies promote expanded implementation.

## Supplementary Information

Below is the link to the electronic supplementary material.

Supplementary file1 (DOCX 85.1 KB)
