# Supplementary material for: Association of Hospital-Based Social Needs Interventions with Potentially Preventable Admissions
Source: J Gen Intern Med. 2025 Jan 17;40(10):2198–206. doi: 10.1007/s11606-024-09203-w (PMC12344029; doi:10.1007/s11606-024-09203-w)
Supplement: Supplementary file 1 — Supplementary file1 (DOCX 85.1 KB) [file 11606_2024_9203_MOESM1_ESM.docx]

**Supplemental Appendices for**

***Association of Hospital-Based Social Needs Interventions with Potentially Preventable Admissions***

Contents

[**Appendix Table 1:** Characteristics of hospitals that provide vs. do not provide each social need intervention 2](#_Toc182997136)

[**Appendix Table 2:** Balance in patient characteristics for each health-related social need activity, before and after weighting 3](#_Toc182997137)

[**Appendix Table 3**: Associations between potentially preventable hospitalizations and each hospital-based activity to address health related social needs (with odds ratios for all covariates modeled) 6](#_Toc182997138)

[**Appendix Table 4:** Social need interventions: implementation & combinations across hospitals in the study sample 14](#_Toc182997139)

[**Appendix Table 5:** Post-hoc sensitivity analyses for potentially preventable admissions based on the chronic conditions composite & diabetes composite measures when hospitals offer meal delivery 15](#_Toc182997140)

[**Appendix 6:** AHA hospital survey questions on health-related social needs interventions 16](#_Toc182997141)

## Appendix Table 1. Characteristics of hospitals that provide vs. do not provide each social need intervention

|  | **Total** | **Meal Delivery** | | | **Transportation** | | | **Mobile Clinics** | | | | **Violence Prevention** | | | |
| --- | --- | --- | --- | --- | --- | --- | --- | --- | --- | --- | --- | --- | --- | --- | --- |
|  |  | **Not Provided** | **Provided** | ***p-value*** | **Not Provided** | **Provided** | ***p-value*** | **Not Provided** | **Provided** | ***p-value*** | **Not Provided** | | **Provided** | ***p-value*** |  |
|  | **N=813** | **N=744** | **N=69** |  | **N=617** | **N=196** |  | **N=681** | **N=132** |  | **N=680** | | **N=133** |  |  |
| Ownership |  |  |  |  |  |  |  |  |  |  |  | |  |  |  |
| Public (county, state, other gov't) | 111 (13.7%) | 108 (14.5%) | 3 (4.3%) | *0.02* | 84 (13.6%) | 27 (13.8%) | *0.07* | 90 (13.2%) | 21 (15.9%) | *0.03* | 96 (14.1%) | | 15 (11.3%) | *0.04* |  |
| Not-for-profit (non  gov't) | 637 (78.4%) | 574 (77.2%) | 63 (91.3%) |  | 476 (77.1%) | 161 (82.1%) |  | 529 (77.7%) | 108 (81.8%) |  | 523 (76.9%) | | 114 (85.7%) |  |  |
| For-profit | 65 (8.0%) | 62 (8.3%) | 3 (4.3%) |  | 57 (9.2%) | 8 (4.1%) |  | 62 (9.1%) | 3 (2.3%) |  | 61 (9.0%) | | 4 (3.0%) |  |  |
| Bed Size |  |  |  |  |  |  |  |  |  |  |  | |  |  |  |
| Up to 99 beds | 322 (39.6%) | 298 (40.1%) | 24 (34.8%) | *0.42* | 276 (44.7%) | 46 (23.5%) | *<0.001* | 307 (45.1%) | 15 (11.4%) | *<0.001* | 296 (43.5%) | | 26 (19.5%) | *<0.001* |  |
| 100 to 299 beds | 283 (34.8%) | 254 (34.1%) | 29 (42.0%) |  | 208 (33.7%) | 75 (38.3%) |  | 240 (35.2%) | 43 (32.6%) |  | 239 (35.1%) | | 44 (33.1%) |  |  |
| 300+ beds | 208 (25.6%) | 192 (25.8%) | 16 (23.2%) |  | 133 (21.6%) | 75 (38.3%) |  | 134 (19.7%) | 74 (56.1%) |  | 145 (21.3%) | | 63 (47.4%) |  |  |
| Member of Larger Health System | 570 (70.1%) | 521 (70.0%) | 49 (71.0%) | *0.86* | 431 (69.9%) | 139 (70.9%) | *0.78* | 473 (69.5%) | 97 (73.5%) | *0.35* | 475 (69.9%) | | 95 (71.4%) | *0.72* |  |
| Teaching Hospital | 63 ( 7.7%) | 56 ( 7.5%) | 7 (10.1%) | *0.44* | 32 ( 5.2%) | 31 (15.8%) | *<0.001* | 34 ( 5.0%) | 29 (22.0%) | *<0.001* | 26 ( 3.8%) | | 37 (27.8%) | *<0.001* |  |
| Participates in an ACO | 360 (44.3%) | 334 (44.9%) | 26 (37.7%) | *0.25* | 245 (39.7%) | 115 (58.7%) | *<0.001* | 275 (40.4%) | 85 (64.4%) | *<0.001* | 288 (42.4%) | | 72 (54.1%) | *0.012* |  |
| Participates in Bundled Payment | 205 (25.2%) | 185 (24.9%) | 20 (29.0%) | *0.45* | 137 (22.2%) | 68 (34.7%) | *<0.001* | 151 (22.2%) | 54 (40.9%) | *<0.001* | 151 (22.2%) | | 54 (40.6%) | *<0.001* |  |
| Rurality |  |  |  |  |  |  |  |  |  |  |  | |  |  |  |
| Metro | 550 (67.7%) | 508 (68.3%) | 42 (60.9%) | *0.17* | 391 (63.4%) | 159 (81.1%) | *<0.001* | 431 (63.3%) | 119 (90.2%) | *<0.001* | 437 (64.3%) | | 113 (85.0%) | *<0.001* |  |
| Micro | 125 (15.4%) | 109 (14.7%) | 16 (23.2%) |  | 106 (17.2%) | 19 (9.7%) |  | 115 (16.9%) | 10 (7.6%) |  | 110 (16.2%) | | 15 (11.3%) |  |  |
| Rural | 138 (17.0%) | 127 (17.1%) | 11 (15.9%) |  | 120 (19.4%) | 18 (9.2%) |  | 135 (19.8%) | 3 (2.3%) |  | 133 (19.6%) | | 5 (3.8%) |  |  |

## Appendix Table 2. Balance in patient characteristics for each health-related social need activity, before and after weighting

| **A. Meal Delivery** | | | | | | | |
| --- | --- | --- | --- | --- | --- | --- | --- |
|  | **Unweighted** | |  |  | **Weighted** |  |  |
|  | Mean in treated | Mean in Untreated | Standardized difference |  | Mean in treated | Mean in Untreated | Standardized difference |
| Sex: Female | 0.51 | 0.51 | 0.006 |  | 0.51 | 0.51 | 0.001 |
| Age: 19to39 | 0.13 | 0.13 | -0.009 |  | 0.14 | 0.13 | 0.012 |
| Age: 40to64 | 0.36 | 0.37 | -0.028 |  | 0.37 | 0.37 | -0.009 |
| Age: 65plus | 0.51 | 0.49 | 0.033 |  | 0.50 | 0.50 | 0.001 |
| Race: White | 0.74 | 0.68 | 0.132 |  | 0.70 | 0.68 | 0.044 |
| Race: Black | 0.12 | 0.15 | -0.064 |  | 0.13 | 0.14 | -0.028 |
| Race: Hispanic | 0.07 | 0.10 | -0.133 |  | 0.09 | 0.10 | -0.034 |
| Race: Asian | 0.02 | 0.02 | 0.004 |  | 0.02 | 0.02 | -0.007 |
| Race: Other | 0.05 | 0.05 | -0.001 |  | 0.05 | 0.05 | 0.003 |
| Comorbidities: 0 | 0.30 | 0.29 | 0.028 |  | 0.29 | 0.29 | 0.000 |
| Comorbidities: 1 | 0.22 | 0.22 | 0.002 |  | 0.22 | 0.22 | -0.002 |
| Comorbidities: 2 | 0.16 | 0.16 | -0.003 |  | 0.16 | 0.16 | 0.002 |
| Comorbidities: 3+ | 0.32 | 0.33 | -0.026 |  | 0.33 | 0.33 | 0.000 |
| Zip code income: low | 0.20 | 0.28 | -0.199 |  | 0.33 | 0.27 | 0.134 |
| Zip code income: low-mid | 0.18 | 0.26 | -0.177 |  | 0.22 | 0.25 | -0.081 |
| Zip code income: mid-high | 0.22 | 0.25 | -0.076 |  | 0.19 | 0.25 | -0.142 |
| Zip code income: high | 0.40 | 0.21 | 0.422 |  | 0.26 | 0.22 | 0.081 |
| Payer: Medicare | 0.55 | 0.54 | 0.017 |  | 0.54 | 0.54 | 0.007 |
| Payer: Medicaid | 0.15 | 0.17 | -0.046 |  | 0.17 | 0.17 | 0.004 |
| Payer: Private | 0.25 | 0.23 | 0.038 |  | 0.23 | 0.23 | -0.011 |
| Payer: Other | 0.06 | 0.06 | -0.032 |  | 0.06 | 0.06 | -0.001 |

| **B. Transportation to Health Services** | | | | | | | |
| --- | --- | --- | --- | --- | --- | --- | --- |
|  | **Unweighted** | |  |  | **Weighted** |  |  |
|  | Mean in treated | Mean in Untreated | Standardized difference |  | Mean in treated | Mean in Untreated | Standardized difference |
| Sex: Female | 0.51 | 0.51 | -0.009 |  | 0.51 | 0.51 | -0.001 |
| Age: 19to39 | 0.14 | 0.13 | 0.040 |  | 0.13 | 0.13 | 0.002 |
| Age: 40to64 | 0.38 | 0.37 | 0.021 |  | 0.37 | 0.37 | -0.003 |
| Age: 65plus | 0.48 | 0.50 | -0.048 |  | 0.50 | 0.50 | 0.001 |
| Race: White | 0.59 | 0.74 | -0.319 |  | 0.68 | 0.68 | -0.002 |
| Race: Black | 0.18 | 0.12 | 0.167 |  | 0.15 | 0.14 | 0.001 |
| Race: Hispanic | 0.13 | 0.08 | 0.142 |  | 0.10 | 0.10 | 0.001 |
| Race: Asian | 0.03 | 0.02 | 0.079 |  | 0.02 | 0.02 | 0.000 |
| Race: Other | 0.07 | 0.03 | 0.162 |  | 0.05 | 0.04 | 0.022 |
| Comorbidities: 0 | 0.30 | 0.28 | 0.026 |  | 0.29 | 0.29 | 0.004 |
| Comorbidities: 1 | 0.21 | 0.22 | -0.005 |  | 0.21 | 0.22 | -0.007 |
| Comorbidities: 2 | 0.16 | 0.16 | -0.007 |  | 0.16 | 0.16 | 0.001 |
| Comorbidities: 3+ | 0.33 | 0.33 | -0.014 |  | 0.33 | 0.33 | 0.001 |
| Zip code income: low | 0.28 | 0.27 | 0.021 |  | 0.29 | 0.27 | 0.042 |
| Zip code income: low-mid | 0.23 | 0.26 | -0.080 |  | 0.24 | 0.26 | -0.037 |
| Zip code income: mid-high | 0.23 | 0.25 | -0.057 |  | 0.23 | 0.26 | -0.071 |
| Zip code income: high | 0.26 | 0.21 | 0.118 |  | 0.25 | 0.22 | 0.065 |
| Payer: Medicare | 0.52 | 0.55 | -0.078 |  | 0.53 | 0.54 | -0.024 |
| Payer: Medicaid | 0.18 | 0.16 | 0.075 |  | 0.17 | 0.16 | 0.018 |
| Payer: Private | 0.24 | 0.23 | 0.027 |  | 0.24 | 0.23 | 0.021 |
| Payer: Other | 0.06 | 0.06 | -0.004 |  | 0.06 | 0.06 | -0.015 |

| **C. Mobile Clinic Services** | | | | | | | |
| --- | --- | --- | --- | --- | --- | --- | --- |
|  | **Unweighted** | |  |  | **Weighted** |  |  |
|  | Mean in treated | Mean in Untreated | Standardized difference |  | Mean in treated | Mean in Untreated | Standardized difference |
| Sex: Female | 0.50 | 0.52 | -0.029 |  | 0.51 | 0.51 | 0.000 |
| Age: 19to39 | 0.15 | 0.12 | 0.072 |  | 0.13 | 0.13 | -0.006 |
| Age: 40to64 | 0.39 | 0.36 | 0.058 |  | 0.38 | 0.37 | 0.011 |
| Age: 65plus | 0.46 | 0.51 | -0.106 |  | 0.49 | 0.50 | -0.006 |
| Race: White | 0.59 | 0.73 | -0.284 |  | 0.68 | 0.68 | -0.003 |
| Race: Black | 0.18 | 0.13 | 0.136 |  | 0.15 | 0.14 | 0.002 |
| Race: Hispanic | 0.13 | 0.08 | 0.163 |  | 0.10 | 0.10 | 0.001 |
| Race: Asian | 0.02 | 0.02 | 0.010 |  | 0.02 | 0.02 | 0.000 |
| Race: Other | 0.07 | 0.04 | 0.153 |  | 0.05 | 0.04 | 0.018 |
| Comorbidities: 0 | 0.29 | 0.29 | 0.014 |  | 0.29 | 0.29 | 0.008 |
| Comorbidities: 1 | 0.21 | 0.22 | -0.021 |  | 0.21 | 0.22 | -0.013 |
| Comorbidities: 2 | 0.16 | 0.16 | -0.006 |  | 0.16 | 0.16 | 0.000 |
| Comorbidities: 3+ | 0.33 | 0.33 | 0.009 |  | 0.33 | 0.33 | 0.003 |
| Zip code income: low | 0.29 | 0.27 | 0.055 |  | 0.28 | 0.27 | 0.035 |
| Zip code income: low-mid | 0.23 | 0.26 | -0.073 |  | 0.24 | 0.26 | -0.046 |
| Zip code income: mid-high | 0.23 | 0.25 | -0.058 |  | 0.23 | 0.25 | -0.057 |
| Zip code income: high | 0.25 | 0.22 | 0.075 |  | 0.25 | 0.22 | 0.067 |
| Payer: Medicare | 0.50 | 0.56 | -0.109 |  | 0.53 | 0.54 | -0.025 |
| Payer: Medicaid | 0.19 | 0.16 | 0.086 |  | 0.17 | 0.16 | 0.009 |
| Payer: Private | 0.24 | 0.23 | 0.018 |  | 0.23 | 0.23 | 0.002 |
| Payer: Other | 0.07 | 0.06 | 0.056 |  | 0.07 | 0.06 | 0.034 |

| **D. Community-Oriented Violence Prevention Programs** | | | | | | | |
| --- | --- | --- | --- | --- | --- | --- | --- |
|  | **Unweighted** | |  |  | **Weighted** |  |  |
|  | Mean in treated | Mean in Untreated | Standardized difference |  | Mean in treated | Mean in Untreated | Standardized difference |
| Sex: Female | 0.51 | 0.51 | -0.013 |  | 0.51 | 0.51 | -0.002 |
| Age: 19to39 | 0.14 | 0.13 | 0.050 |  | 0.13 | 0.13 | 0.000 |
| Age: 40to64 | 0.39 | 0.37 | 0.040 |  | 0.38 | 0.37 | 0.013 |
| Age: 65plus | 0.47 | 0.51 | -0.073 |  | 0.49 | 0.50 | -0.012 |
| Race: White | 0.60 | 0.72 | -0.243 |  | 0.68 | 0.68 | -0.011 |
| Race: Black | 0.17 | 0.13 | 0.095 |  | 0.15 | 0.15 | 0.008 |
| Race: Hispanic | 0.12 | 0.09 | 0.114 |  | 0.10 | 0.10 | 0.005 |
| Race: Asian | 0.03 | 0.02 | 0.034 |  | 0.02 | 0.02 | 0.004 |
| Race: Other | 0.08 | 0.03 | 0.185 |  | 0.05 | 0.04 | 0.021 |
| Comorbidities: 0 | 0.29 | 0.29 | 0.002 |  | 0.29 | 0.29 | 0.010 |
| Comorbidities: 1 | 0.21 | 0.22 | -0.023 |  | 0.21 | 0.22 | -0.012 |
| Comorbidities: 2 | 0.16 | 0.16 | 0.000 |  | 0.16 | 0.16 | 0.001 |
| Comorbidities: 3+ | 0.34 | 0.33 | 0.018 |  | 0.33 | 0.33 | 0.001 |
| Zip code income: low | 0.28 | 0.28 | 0.000 |  | 0.30 | 0.26 | 0.089 |
| Zip code income: low-mid | 0.20 | 0.27 | -0.163 |  | 0.22 | 0.26 | -0.110 |
| Zip code income: mid-high | 0.23 | 0.25 | -0.049 |  | 0.22 | 0.26 | -0.081 |
| Zip code income: high | 0.29 | 0.20 | 0.211 |  | 0.26 | 0.22 | 0.096 |
| Payer: Medicare | 0.51 | 0.55 | -0.084 |  | 0.53 | 0.54 | -0.026 |
| Payer: Medicaid | 0.19 | 0.16 | 0.083 |  | 0.18 | 0.16 | 0.041 |
| Payer: Private | 0.25 | 0.23 | 0.048 |  | 0.24 | 0.23 | 0.021 |
| Payer: Other | 0.06 | 0.07 | -0.040 |  | 0.05 | 0.07 | -0.049 |

## Appendix Table 3 Associations between potentially preventable hospitalizations and each hospital-based activity to address health related social needs (with odds ratios for all covariates modeled)

| 1. **Meal Delivery** | | | | | | |
| --- | --- | --- | --- | --- | --- | --- |
|  | **Full sample** | | **Medicaid Only** | | **Medicare Only** | |
|  | Odds Ratio | 95% Confidence Interval | Odds Ratio | 95% Confidence Interval | Odds Ratio | 95% Confidence Interval |
| Meal Delivery | 0.905* | [0.825,0.993] | 0.794*** | [0.697,0.904] | 0.931 | [0.851,1.019] |
| Sex: Female | 1.129*** | [1.118,1.140] | 1.133*** | [1.103,1.165] | 1.146*** | [1.135,1.157] |
| Age: 19to39 | 1 | [1,1] | 1 | [1,1] | 1 | [1,1] |
| Age: 40to64 | 0.994 | [0.962,1.026] | 1.005 | [0.967,1.045] | 1.264*** | [1.208,1.323] |
| Age: 65+ | 1.124*** | [1.081,1.169] | 1.279*** | [1.202,1.361] | 1.315*** | [1.251,1.383] |
| Race: White | 1 | [1,1] | 1 | [1,1] | 1 | [1,1] |
| Race: Black | 1.424*** | [1.378,1.471] | 1.461*** | [1.394,1.530] | 1.277*** | [1.237,1.319] |
| Race: Hispanic | 1.174*** | [1.123,1.227] | 1.115*** | [1.048,1.186] | 1.178*** | [1.127,1.233] |
| Race: Asian | 0.957 | [0.855,1.072] | 0.963 | [0.833,1.112] | 0.949 | [0.845,1.067] |
| Race: Other | 0.975 | [0.931,1.021] | 0.99 | [0.928,1.055] | 0.966 | [0.916,1.019] |
| Zip code income: low | 1 | [1,1] | 1 | [1,1] | 1 | [1,1] |
| Zip code income: low-mid | 0.925*** | [0.897,0.953] | 0.923*** | [0.885,0.963] | 0.941*** | [0.913,0.970] |
| Zip code income: mid-high | 0.878*** | [0.849,0.907] | 0.875*** | [0.836,0.917] | 0.902*** | [0.873,0.933] |
| Zip code income: high | 0.865*** | [0.823,0.910] | 0.843*** | [0.787,0.902] | 0.912*** | [0.869,0.957] |
| Comorbidities: 0 | 1 | [1,1] | 1 | [1,1] | 1 | [1,1] |
| Comorbidities: 1 | 4.974*** | [4.837,5.115] | 7.148*** | [6.764,7.553] | 3.727*** | [3.630,3.827] |
| Comorbidities: 2 | 5.929*** | [5.717,6.149] | 9.913*** | [9.261,10.61] | 4.483*** | [4.341,4.630] |
| Comorbidities: 3+ | 7.547*** | [7.259,7.847] | 9.949*** | [9.252,10.70] | 6.087*** | [5.869,6.313] |
| Payer: Medicare | 1 | [1,1] |  |  |  |  |
| Payer: Medicaid | 1.002 | [0.977,1.028] |  |  |  |  |
| Payer: Private | 0.715*** | [0.695,0.735] |  |  |  |  |
| Payer: Other | 0.98 | [0.941,1.020] |  |  |  |  |
| Ownership: Public | 1 | [1,1] | 1 | [1,1] | 1 | [1,1] |
| Ownership: Non-profit | 1.012 | [0.935,1.096] | 1.063 | [0.945,1.196] | 0.979 | [0.907,1.056] |
| Ownership: For-profit | 1.011 | [0.905,1.130] | 1.134 | [0.978,1.316] | 0.98 | [0.878,1.093] |
| Size: 0-99 beds | 1 | [1,1] | 1 | [1,1] | 1 | [1,1] |
| Size: 100-299 beds | 0.795*** | [0.727,0.869] | 0.771*** | [0.681,0.872] | 0.808*** | [0.740,0.882] |
| Size: 300+ beds | 0.714*** | [0.652,0.782] | 0.684*** | [0.601,0.780] | 0.734*** | [0.672,0.802] |
| Part of a system | 1.032 | [0.970,1.098] | 1.089 | [0.995,1.191] | 1.012 | [0.951,1.076] |
| Teaching hospital | 0.805*** | [0.741,0.876] | 0.804*** | [0.739,0.874] | 0.832*** | [0.769,0.901] |
| ACO participant | 0.981 | [0.933,1.033] | 0.995 | [0.934,1.061] | 0.966 | [0.920,1.015] |
| Bundled payment participant | 0.997 | [0.942,1.057] | 1.017 | [0.952,1.085] | 0.989 | [0.937,1.044] |
| Rurality: Metropolitan | 1 | [1,1] | 1 | [1,1] | 1 | [1,1] |
| Rurality: Micropolitan | 1.175*** | [1.069,1.292] | 1.162* | [1.029,1.312] | 1.159** | [1.060,1.267] |
| Rurality: Rural | 1.736*** | [1.422,2.118] | 1.462** | [1.116,1.915] | 1.754*** | [1.452,2.119] |

* = p < 0.05; ** = p< 0.01 *** = p<0.001

| 1. **Transportation to Medical Appointments** | | | | | | |
| --- | --- | --- | --- | --- | --- | --- |
|  | **Full sample** | | **Medicaid Only** | | **Medicare Only** | |
|  | Odds Ratio | 95% Confidence Interval | Odds Ratio | 95% Confidence Interval | Odds Ratio | 95% Confidence Interval |
| Transportation | 1.024 | [0.966,1.087] | 0.969 | [0.900,1.043] | 1.031 | [0.974,1.091] |
| Sex: Female | 1.128*** | [1.117,1.139] | 1.130*** | [1.100,1.161] | 1.146*** | [1.135,1.157] |
| Age: 19to39 | 1 | [1,1] | 1 | [1,1] | 1 | [1,1] |
| Age: 40to64 | 0.991 | [0.960,1.023] | 1.002 | [0.965,1.041] | 1.256*** | [1.200,1.315] |
| Age: 65+ | 1.120*** | [1.077,1.164] | 1.272*** | [1.192,1.357] | 1.306*** | [1.242,1.374] |
| Race: White | 1 | [1,1] | 1 | [1,1] | 1 | [1,1] |
| Race: Black | 1.418*** | [1.373,1.464] | 1.472*** | [1.408,1.538] | 1.270*** | [1.230,1.310] |
| Race: Hispanic | 1.168*** | [1.115,1.224] | 1.117*** | [1.053,1.185] | 1.170*** | [1.115,1.228] |
| Race: Asian | 0.933 | [0.851,1.023] | 0.941 | [0.823,1.077] | 0.924 | [0.843,1.014] |
| Race: Other | 0.958* | [0.918,0.999] | 0.976 | [0.916,1.039] | 0.951* | [0.906,0.998] |
| Zip code income: low | 1 | [1,1] | 1 | [1,1] | 1 | [1,1] |
| Zip code income: low-mid | 0.924*** | [0.897,0.952] | 0.920*** | [0.882,0.958] | 0.939*** | [0.912,0.968] |
| Zip code income: mid-high | 0.876*** | [0.848,0.905] | 0.867*** | [0.829,0.907] | 0.900*** | [0.871,0.931] |
| Zip code income: high | 0.863*** | [0.822,0.907] | 0.826*** | [0.772,0.885] | 0.912*** | [0.869,0.956] |
| Comorbidities: 0 | 1 | [1,1] | 1 | [1,1] | 1 | [1,1] |
| Comorbidities: 1 | 4.998*** | [4.863,5.136] | 7.234*** | [6.864,7.624] | 3.741*** | [3.646,3.838] |
| Comorbidities: 2 | 5.965*** | [5.752,6.186] | 10.06*** | [9.418,10.75] | 4.504*** | [4.362,4.650] |
| Comorbidities: 3+ | 7.586*** | [7.295,7.889] | 10.09*** | [9.399,10.84] | 6.112*** | [5.895,6.338] |
| Payer: Medicare | 1 | [1,1] |  |  |  |  |
| Payer: Medicaid | 1.004 | [0.980,1.029] |  |  |  |  |
| Payer: Private | 0.714*** | [0.694,0.734] |  |  |  |  |
| Payer: Other | 0.984 | [0.943,1.026] |  |  |  |  |
| Ownership: Public | 1 | [1,1] | 1 | [1,1] | 1 | [1,1] |
| Ownership: Non-profit | 1.002 | [0.927,1.084] | 1.062 | [0.945,1.193] | 0.969 | [0.899,1.044] |
| Ownership: For-profit | 1.004 | [0.899,1.122] | 1.134 | [0.976,1.317] | 0.973 | [0.870,1.087] |
| Size: 0-99 beds | 1 | [1,1] | 1 | [1,1] | 1 | [1,1] |
| Size: 100-299 beds | 0.804*** | [0.740,0.874] | 0.779*** | [0.692,0.878] | 0.817*** | [0.753,0.888] |
| Size: 300+ beds | 0.723*** | [0.663,0.788] | 0.699*** | [0.617,0.792] | 0.743*** | [0.682,0.808] |
| Part of a system | 1.027 | [0.967,1.092] | 1.065 | [0.977,1.162] | 1.01 | [0.950,1.074] |
| Teaching hospital | 0.800*** | [0.732,0.873] | 0.801*** | [0.734,0.875] | 0.825*** | [0.760,0.896] |
| ACO participant | 0.982 | [0.930,1.036] | 1.004 | [0.941,1.071] | 0.965 | [0.917,1.016] |
| Bundled payment participant | 0.995 | [0.938,1.055] | 1.01 | [0.945,1.079] | 0.99 | [0.937,1.046] |
| Rurality: Metropolitan | 1 | [1,1] | 1 | [1,1] | 1 | [1,1] |
| Rurality: Micropolitan | 1.156** | [1.055,1.266] | 1.145* | [1.022,1.282] | 1.143** | [1.047,1.248] |
| Rurality: Rural | 1.774*** | [1.489,2.114] | 1.475** | [1.132,1.922] | 1.799*** | [1.526,2.119] |

* = p < 0.05; ** = p< 0.01 *** = p<0.001

| 1. **Mobile Clinics** | | | | | | |
| --- | --- | --- | --- | --- | --- | --- |
|  | **Full sample** | | **Medicaid Only** | | **Medicare Only** | |
|  | Odds Ratio | 95% Confidence Interval | Odds Ratio | 95% Confidence Interval | Odds Ratio | 95% Confidence Interval |
| Mobile Clinics | 0.991 | [0.931,1.055] | 0.998 | [0.928,1.073] | 0.992 | [0.934,1.054] |
| Sex: Female | 1.129*** | [1.118,1.140] | 1.134*** | [1.103,1.165] | 1.146*** | [1.135,1.158] |
| Age: 19to39 | 1 | [1,1] | 1 | [1,1] | 1 | [1,1] |
| Age: 40to64 | 0.991 | [0.960,1.023] | 1.002 | [0.964,1.041] | 1.257*** | [1.201,1.316] |
| Age: 65+ | 1.119*** | [1.077,1.164] | 1.271*** | [1.195,1.352] | 1.307*** | [1.243,1.374] |
| Race: White | 1 | [1,1] | 1 | [1,1] | 1 | [1,1] |
| Race: Black | 1.421*** | [1.376,1.468] | 1.462*** | [1.394,1.532] | 1.274*** | [1.235,1.315] |
| Race: Hispanic | 1.173*** | [1.125,1.224] | 1.113*** | [1.050,1.180] | 1.178*** | [1.127,1.231] |
| Race: Asian | 0.956 | [0.854,1.072] | 0.963 | [0.829,1.119] | 0.947 | [0.843,1.063] |
| Race: Other | 0.965 | [0.924,1.008] | 0.972 | [0.913,1.035] | 0.955 | [0.910,1.003] |
| Zip code income: low | 1 | [1,1] | 1 | [1,1] | 1 | [1,1] |
| Zip code income: low-mid | 0.926*** | [0.898,0.954] | 0.924*** | [0.886,0.963] | 0.942*** | [0.914,0.970] |
| Zip code income: mid-high | 0.878*** | [0.849,0.907] | 0.872*** | [0.834,0.913] | 0.903*** | [0.874,0.933] |
| Zip code income: high | 0.867*** | [0.826,0.911] | 0.839*** | [0.784,0.899] | 0.915*** | [0.873,0.959] |
| Comorbidities: 0 | 1 | [1,1] | 1 | [1,1] | 1 | [1,1] |
| Comorbidities: 1 | 4.986*** | [4.848,5.127] | 7.191*** | [6.810,7.593] | 3.735*** | [3.637,3.836] |
| Comorbidities: 2 | 5.959*** | [5.745,6.181] | 10.03*** | [9.370,10.75] | 4.500*** | [4.358,4.646] |
| Comorbidities: 3+ | 7.581*** | [7.289,7.885] | 10.10*** | [9.387,10.86] | 6.108*** | [5.890,6.334] |
| Payer: Medicare | 1 | [1,1] |  |  |  |  |
| Payer: Medicaid | 1.004 | [0.979,1.029] |  |  |  |  |
| Payer: Private | 0.715*** | [0.695,0.735] |  |  |  |  |
| Payer: Other | 0.977 | [0.940,1.017] |  |  |  |  |
| Ownership: Public | 1 | [1,1] | 1 | [1,1] | 1 | [1,1] |
| Ownership: Non-profit | 1.006 | [0.929,1.089] | 1.056 | [0.941,1.185] | 0.976 | [0.904,1.055] |
| Ownership: For-profit | 1.005 | [0.899,1.123] | 1.131 | [0.976,1.311] | 0.976 | [0.873,1.091] |
| Size: 0-99 beds | 1 | [1,1] | 1 | [1,1] | 1 | [1,1] |
| Size: 100-299 beds | 0.807*** | [0.742,0.877] | 0.782*** | [0.690,0.887] | 0.820*** | [0.756,0.890] |
| Size: 300+ beds | 0.724*** | [0.664,0.789] | 0.697*** | [0.609,0.797] | 0.745*** | [0.684,0.810] |
| Part of a system | 1.032 | [0.971,1.097] | 1.076 | [0.982,1.179] | 1.015 | [0.955,1.078] |
| Teaching hospital | 0.805*** | [0.740,0.876] | 0.798*** | [0.732,0.871] | 0.835*** | [0.771,0.904] |
| ACO participant | 0.986 | [0.936,1.039] | 1.003 | [0.941,1.070] | 0.97 | [0.921,1.021] |
| Bundled payment participant | 0.993 | [0.938,1.052] | 1.011 | [0.946,1.079] | 0.988 | [0.935,1.044] |
| Rurality: Metropolitan | 1 | [1,1] | 1 | [1,1] | 1 | [1,1] |
| Rurality: Micropolitan | 1.158** | [1.061,1.265] | 1.148* | [1.023,1.289] | 1.146** | [1.053,1.248] |
| Rurality: Rural | 1.763*** | [1.476,2.105] | 1.467** | [1.121,1.919] | 1.791*** | [1.518,2.113] |

* = p < 0.05; ** = p< 0.01 *** = p<0.001

| 1. **Community-Oriented Violence Prevention Programs** | | | | | | |
| --- | --- | --- | --- | --- | --- | --- |
|  | **Full sample** | | **Medicaid Only** | | **Medicare Only** | |
|  | Odds Ratio | 95% Confidence Interval | Odds Ratio | 95% Confidence Interval | Odds Ratio | 95% Confidence Interval |
| Violence Prevention | 0.958 | [0.898,1.022] | 0.962 | [0.895,1.034] | 0.978 | [0.918,1.041] |
| Sex: Female | 1.129*** | [1.118,1.140] | 1.134*** | [1.103,1.165] | 1.146*** | [1.135,1.158] |
| Age: 19to39 | 1 | [1,1] | 1 | [1,1] | 1 | [1,1] |
| Age: 40to64 | 0.991 | [0.960,1.023] | 1.002 | [0.964,1.041] | 1.257*** | [1.201,1.316] |
| Age: 65+ | 1.119*** | [1.077,1.164] | 1.271*** | [1.195,1.352] | 1.307*** | [1.243,1.374] |
| Race: White | 1 | [1,1] | 1 | [1,1] | 1 | [1,1] |
| Race: Black | 1.421*** | [1.376,1.468] | 1.462*** | [1.394,1.532] | 1.274*** | [1.235,1.315] |
| Race: Hispanic | 1.173*** | [1.125,1.224] | 1.113*** | [1.050,1.180] | 1.178*** | [1.127,1.231] |
| Race: Asian | 0.956 | [0.854,1.072] | 0.963 | [0.829,1.119] | 0.947 | [0.843,1.063] |
| Race: Other | 0.965 | [0.924,1.008] | 0.972 | [0.913,1.035] | 0.955 | [0.910,1.003] |
| Zip code income: low | 1 | [1,1] | 1 | [1,1] | 1 | [1,1] |
| Zip code income: low-mid | 0.926*** | [0.898,0.954] | 0.924*** | [0.886,0.963] | 0.942*** | [0.914,0.970] |
| Zip code income: mid-high | 0.878*** | [0.849,0.907] | 0.872*** | [0.834,0.913] | 0.903*** | [0.874,0.933] |
| Zip code income: high | 0.867*** | [0.826,0.911] | 0.839*** | [0.784,0.899] | 0.915*** | [0.873,0.959] |
| Comorbidities: 0 | 1 | [1,1] | 1 | [1,1] | 1 | [1,1] |
| Comorbidities: 1 | 4.986*** | [4.848,5.127] | 7.191*** | [6.810,7.593] | 3.735*** | [3.637,3.836] |
| Comorbidities: 2 | 5.959*** | [5.745,6.181] | 10.03*** | [9.370,10.75] | 4.500*** | [4.358,4.646] |
| Comorbidities: 3+ | 7.581*** | [7.289,7.885] | 10.10*** | [9.387,10.86] | 6.108*** | [5.890,6.334] |
| Payer: Medicare | 1 | [1,1] |  |  |  |  |
| Payer: Medicaid | 1.004 | [0.979,1.029] |  |  |  |  |
| Payer: Private | 0.715*** | [0.695,0.735] |  |  |  |  |
| Payer: Other | 0.977 | [0.940,1.017] |  |  |  |  |
| Ownership: Public | 1 | [1,1] | 1 | [1,1] | 1 | [1,1] |
| Ownership: Non-profit | 1.006 | [0.929,1.089] | 1.056 | [0.941,1.185] | 0.976 | [0.904,1.055] |
| Ownership: For-profit | 1.005 | [0.899,1.123] | 1.131 | [0.976,1.311] | 0.976 | [0.873,1.091] |
| Size: 0-99 beds | 1 | [1,1] | 1 | [1,1] | 1 | [1,1] |
| Size: 100-299 beds | 0.807*** | [0.742,0.877] | 0.782*** | [0.690,0.887] | 0.820*** | [0.756,0.890] |
| Size: 300+ beds | 0.724*** | [0.664,0.789] | 0.697*** | [0.609,0.797] | 0.745*** | [0.684,0.810] |
| Part of a system | 1.032 | [0.971,1.097] | 1.076 | [0.982,1.179] | 1.015 | [0.955,1.078] |
| Teaching hospital | 0.805*** | [0.740,0.876] | 0.798*** | [0.732,0.871] | 0.835*** | [0.771,0.904] |
| ACO participant | 0.986 | [0.936,1.039] | 1.003 | [0.941,1.070] | 0.97 | [0.921,1.021] |
| Bundled payment participant | 0.993 | [0.938,1.052] | 1.011 | [0.946,1.079] | 0.988 | [0.935,1.044] |
| Rurality: Metropolitan | 1 | [1,1] | 1 | [1,1] | 1 | [1,1] |
| Rurality: Micropolitan | 1.158** | [1.061,1.265] | 1.148* | [1.023,1.289] | 1.146** | [1.053,1.248] |
| Rurality: Rural | 1.763*** | [1.476,2.105] | 1.467** | [1.121,1.919] | 1.791*** | [1.518,2.113] |

* = p < 0.05; ** = p< 0.01 *** = p<0.001

## Appendix Table 4: Social need interventions: implementation & combinations across hospitals in the study sample

|  | **N (hospitals)** | **%** |
| --- | --- | --- |
| **None of the social need interventions** | **449** | **55%** |
|  |  |  |
| **1 intervention only** | **243** | **30%** |
| Meals only | 37 | 5% |
| Transportation only | 98 | 12% |
| Mobile clinic only | 53 | 7% |
| Violence prevention only | 55 | 7% |
|  |  |  |
| **2 interventions only** | **83** | **10%** |
| Meals + transportation only | 4 | 0% |
| Meals + mobile clinic only | 1 | 0% |
| Meals + violence prevention only | 3 | 0% |
| Transportation + mobile clinic only | 30 | 4% |
| Transportation + violence prevention only | 28 | 3% |
| Mobile clinic + violence prevention only | 17 | 2% |
|  |  |  |
| **3 interventions only** | **31** | **4%** |
| Meals + transportation + mobile clinic | 8 | 1% |
| Meals + mobile clinic + violence prevention | 2 | 0% |
| Transportation + mobile clinic + violence prevention | 14 | 2% |
| Transportation + meals + violence prevention | 7 | 1% |
|  |  |  |
| **All 4 interventions** (Meals + transportation + mobile clinic + violence prevention) | **7** | **1%** |
|  |  |  |
| **Total N (hospitals)** | **813** | **100%** |

## Appendix Table 5: Post-hoc sensitivity analyses for potentially preventable admissions based on the chronic conditions composite & diabetes composite measures when hospitals offer meal delivery

| **GLM Models** | **Overall** | | | **Medicaid** | | | **Medicare** | | |
| --- | --- | --- | --- | --- | --- | --- | --- | --- | --- |
|  | **Marginal Effect** | **95% CI LL** | **95% CI UL** | **Marginal Effect** | **95% CI LL** | **95% CI UL** | **Marginal Effect** | **95% CI LL** | **95% CI UL** |
| Main Analysis: Overall Composite (PQI #90) | -1.1 | -2.1 | -0.1 | -2.3 | -3.5 | -1.0 | -0.9 | -2.1 | 0.2 |
| Chronic Conditions Admissions (PQI #92) | -0.7 | -1.4 | -0.1 | -1.8 | -2.7 | -0.9 | -0.5 | -1.3 | 0.2 |
| Diabetes-Composite Admissions (PQI #93) | -0.3 | -0.6 | 0.0 | -1.0 | -1.6 | -0.4 | -0.1 | -0.3 | 0.1 |

*All models control for gender, age category, race, zip code level income, comorbidities, payer, ownership, beds, system, teaching, accountable care organization participation, bundled payment participation, and rurality.

PQI = Prevention Quality Indicator; CI = confidence interval; LL = lower limit; UL = upper limit

## Appendix 6: AHA hospital survey questions on health-related social needs interventions

For each service or facility listed below, please check all the categories that describe how each item is provided **as of the last day of the reporting period**. Check all categories that apply for an item.

| **Service** | **Owned or provided by my hospital or its subsidiary** | **Provided by my health system (in my local community)** | **Provided through a formal contractual arrangement or joint venture with another provider that is not in my system (in my local community)** | **Do not provide** |
| --- | --- | --- | --- | --- |
| 66. Meals on Wheels | 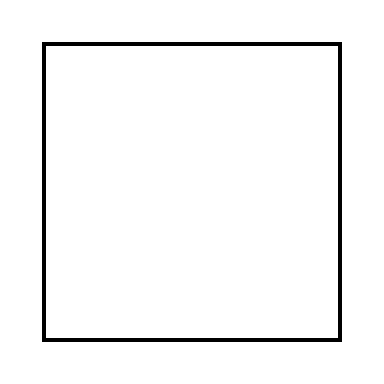 | 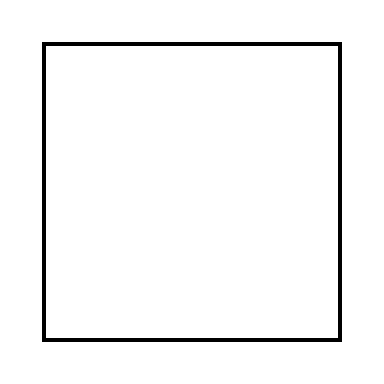 | 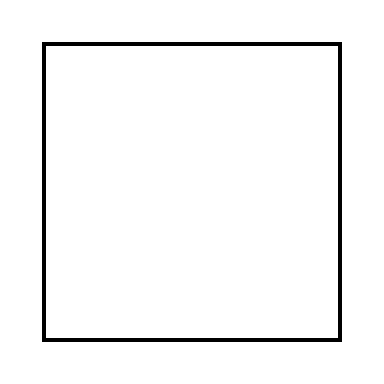 | 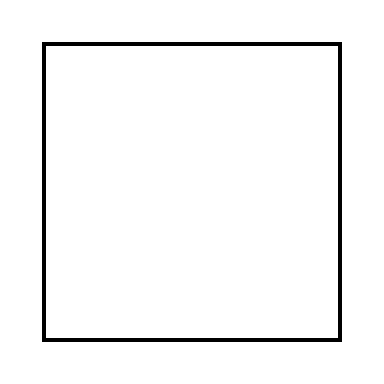 |
| 67. Mobile health services | 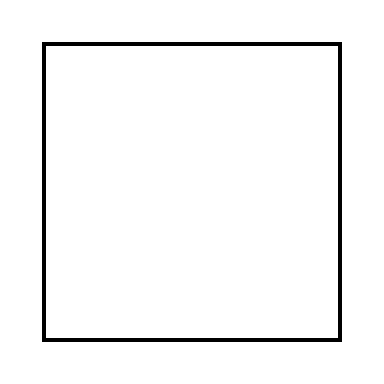 | 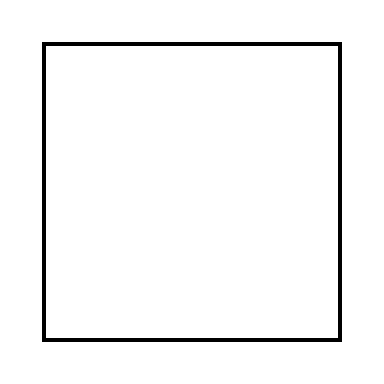 | 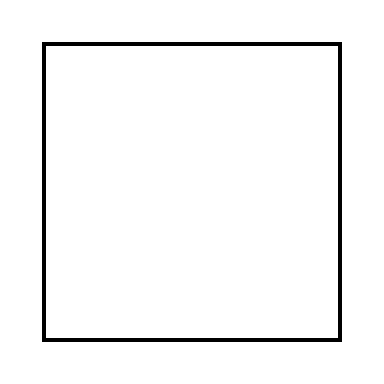 | 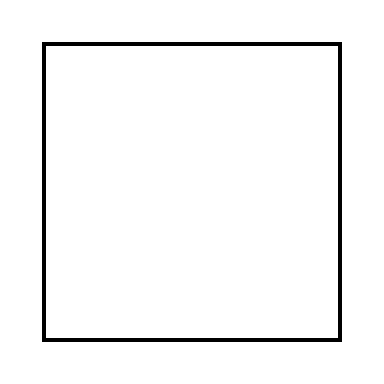 |
| 97. Transportation to health services | 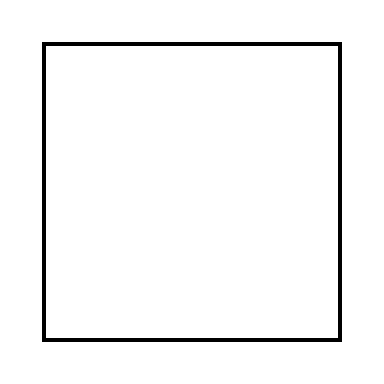 | 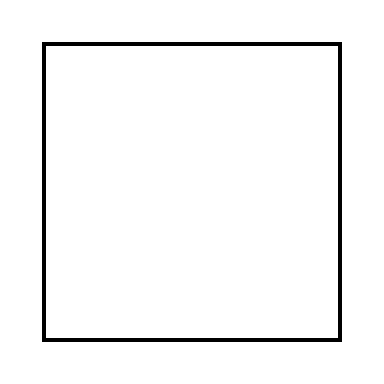 | 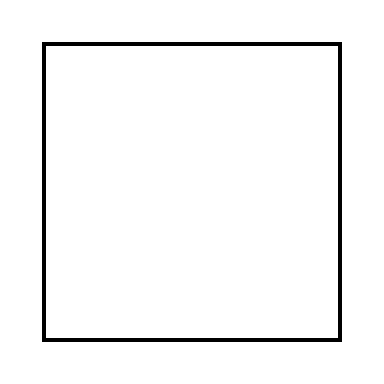 | 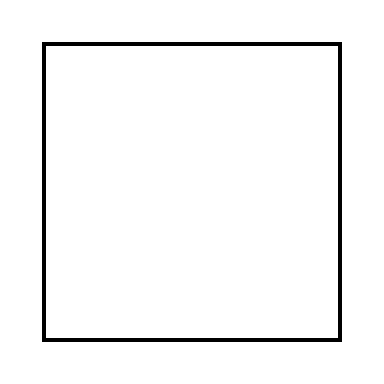 |
| 99b. Violence prevention programs for the community | 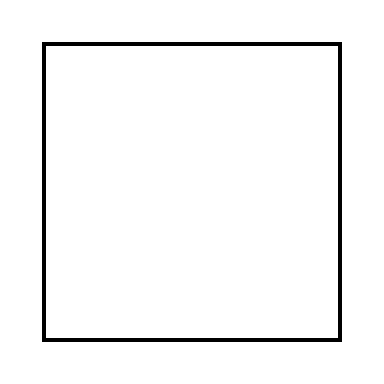 | 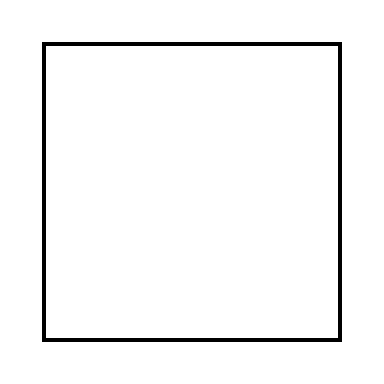 | 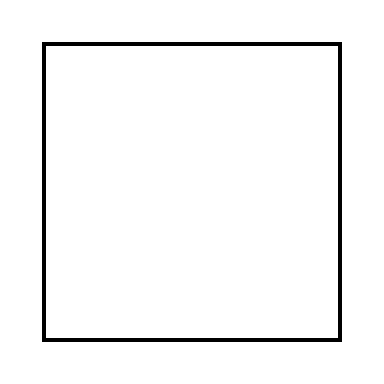 | 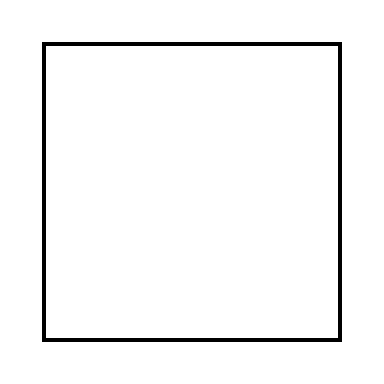 |

***[Note: “Owned or provided by my hospital or its subsidiary” response was used to define if intervention was implemented.]***

**Survey Definitions:**

**Meals on wheels.** A hospital sponsored program which delivers meals to people, usually the elderly, who are unable to prepare their own meals. Low cost, nutritional meals are delivered to individuals’ homes on a regular basis.

**Mobile health services.** Vans and other vehicles used for delivery to primary care services.

**Transportation to health facilities.** A long-term care support service designed to assist the mobility of the elderly. Some programs offer improved financial access by offering reduced rates and barrier-free buses or vans with ramps and lifts to assist the elderly or people with disabilities; others offer subsidies for public transport systems or operate mini-bus services exclusively for use by senior citizens.

**Violence Prevention [Community].** An organized program that attempts to make a positive impact on the type(s) of violence a community is experiencing. For example, it can assist victims of violent crimes, e.g., rape, or incidents, e.g., bullying, to hospital or to community services to prevent further victimization or retaliation. A program that targets the underlying circumstances that contribute to violence such as poor housing, insufficient job training, and/or substance abuse through means such direct involvement and support, education, mentoring, anger management, crisis intervention and training programs would also qualify.
